# Innovative information-sharing strategies.

**DOI:** 10.3201/eid0403.980334

**Published:** 1998

**Authors:** B A Kay, R J Timperi, S S Morse, D Forslund, J J McGowan, T O'Brien

**Affiliations:** Centers for Disease Control and Prevention, Atlanta, Georgia, USA.


### Innovative Information-Sharing Strategies

**Bradford A. Kay,* Ralph J. Timperi,† Stephen S. Morse,‡ David Forslund,§ Julie J. McGowan,¶ and Thomas O'Brien#**
*Centers for Disease Control and Prevention, Atlanta, Georgia, USA; †The Massachusetts State Laboratory Institute, Jamaica Plain, Massachusetts, USA; ‡Defense Advanced Research Projects Agency, Arlington, Virginia, USA; §Los Alamos National Laboratory, Los Alamos, New Mexico, USA; ¶University of Vermont, Burlington, Vermont, USA; and #Brigham and Women's Hospital, Boston, Massachusetts, USA

National and global health issues accentuate the need for health professionals to rapidly and effectively acquire and disseminate information. This session highlighted four innovative systems for communicating health information.

### ProMED

Many experts, both within and outside government, have warned of the need to improve capabilities for dealing with emerging infectious diseases; development of an effective global infectious disease surveillance system has been the primary recommendation. ProMED, a project of the Federation of American Scientists, was inaugurated in 1993 at a conference in Geneva as a vehicle for developing, coordinating, and promoting plans for a global program to identify and respond to emerging infectious diseases. Members of the ProMED Steering Committee include (among others) representatives of the Centers for Disease Control and Prevention, the National Institutes of Health, the World Health Organization (WHO), the Pan American Health Organization, and the International Office of Epizootics.

In 1994, in cooperation with SatelLife/HealthNet, ProMED developed an e-mail conference system, ProMED-mail, on the Internet. Originally developed to allow worldwide scientist-to-scientist communications on emerging infectious diseases, the system rapidly evolved into a prototype for an open-architecture, real-time outbreak reporting system intended to complement official surveillance systems. Today, with more than 10,000 subscribers from more than 125 countries, ProMED-mail is increasingly providing the first reports of infectious disease outbreaks. All items are read by scientists before posting. Reporting of incidents or outbreaks, infectious disease problems of emerging interest, and discussions on how to improve surveillance and response capabilities are especially encouraged. To subscribe to the ProMED-mail electronic conference, send an e-mail message to majordomo@usa.healthnet.org, and write "subscribe promed" in the text space.

### TeleMed

The Advanced Computing Laboratory at Los Alamos National Laboratory, Los Alamos, New Mexico, developed TeleMed, an electronic medical record for managing tuberculosis patients through a collaboration with the National Jewish Center for Immunology and Respiratory Medicine in Denver, Colorado. TeleMed provides a snapshot of patient data, presented chronologically with access to laboratory test results, clinical history, radiology images, reports, and treatment history. A particularly valuable feature allows physicians to annotate the medical record, either orally or in writing, for collaborating physicians to retrieve. Medical expertise can also be exchanged in real time, with both users sharing the same screen and with each having the capability to drive the mouse-pointer. TeleMed, now available on the Internet using Java-based technology, enables physician specialists to support primary care providers in the management of complex medical problems. The technology creates a "virtual patient record" that allows the integration of databases from multiple clinics and multiple providers across geographically separated areas. This permits individual health-care facilities to continue to own and manage their own data while making the data accessible to others treating the same patient. TeleMed provides a time-oriented record of the patient's medical history but only retrieves the actual data on demand, thereby minimizing the bandwidth requirements of the networking capabilities. Distributed ownership of the data means that only one copy of the data exists, and that copy remains where it was created. Location of the data is obtained from a master patient index that provides pointers to the data. Security and access to the data are controlled and protected with encryption technology.

### VTMedNet

Vermont MedNet has been described as the "first comprehensive statewide health information network in the nation." VTMedNet was developed to provide timely access to medical information in support of health-care delivery across the state of Vermont. The system was unique, not because it used advanced technology, but because it used basic technology. VTMedNet Plus, the network's evolution into voice, image, and video, has already garnered national recognition for its initiatives in the area of telemedicine. The network's home page has become a primary resource for the dissemination of information about Vermont's health-care community and information for Vermont's health-care consumer. It is also being used to collect data for research and public health reporting and to distribute aggregate information to improve health-care delivery in one of the nation's most rural states. VTMedNet is the culmination of a partnership among all major health-care organizations in Vermont, including the University of Vermont, Fletcher Allen Health Care, Vermont State Medical Society, and the Vermont Hospital Association. To access the network, users must have their own computers and modems. A simple, configured, shareware communications script is provided to those who request it. VTMedNet is primarily an intranet and provides e-mail and Internet access for the state's health-care providers and access to health information from around the world. It is also designed to serve as a "virtual colleague," encouraging communication among all of Vermont's health-care providers through targeted listservs.

### WHONET

WHONET is database software for the management of routine microbiologic test results. Its primary goals are to enhance local capabilities for analysis and to facilitate the exchange of microbiologic data between centers. WHONET is a DOS-based application that may be used alone on personal computers or in conjunction with existing mainframe- or minicomputer-based clinical information systems. Data conversion ("downloading" or "translating") from hospital systems or commercial automated susceptibility test machines can usually be accomplished with BACLINK software, also available free of charge from WHO. WHONET is not a complete laboratory management system but can be used for simple clinical reporting of results. Software development has concentrated on data analysis, particularly of the results of antimicrobial susceptibility tests. The analytic tools aid the selection of antimicrobial agents, the identification of hospital outbreaks, and the recognition of quality control problems in the laboratory. Review of antimicrobial results also permits characterization of resistance mechanisms and the epidemiology of resistant strains.

The software consists of three sections. 1) Data Entry. In addition to the routine entry of susceptibility test results (disk diffusion, MIC, and/or E-test), this program permits printing, retrieval, and correction of clinical records as well as immediate feedback on test results. If data are converted from an existing laboratory system, for example with BACLINK, direct entry of data into WHONET is unnecessary. 2) Data Analysis. Currently supported analyses include listings and summaries of isolates by user-defined criteria; tabulation of the percentages of resistant, intermediate, and susceptible isolates by species; zone diameter and MIC histograms; scatterplots of zone diameter versus zone diameter or MIC versus MIC; scatterplots of zone diameter versus MIC scatterplots and the calculation of zone diameter/MIC regression curves; listings and summaries of isolates by resistance profile; and automated screening of the data for unusual isolates. 3) Configuration Program. This program permits the user to enter and modify laboratory-specific information such as patient-care areas, antibiotics and interpretive breakpoints, language, and hardware.

